# Improving the operational durability of tangential-rotary picks for rock cutting with central spraying and hydrodynamic rotation

**DOI:** 10.1038/s41598-024-63733-1

**Published:** 2024-06-05

**Authors:** Piotr Cheluszka, Stanisław Mikuła, Jarosław Mikuła, Jiří Fries

**Affiliations:** 1 Department of Mining Mechanization and Robotisation, Faculty of Mining, Safety Engineering and Industrial Automation, Silesian University of Technology, Akademicka 2, 44-100 Gliwice, Poland; 2 Department of Engineering Materials and Biomaterials, Faculty of Mechanical Engineering, Silesian University of Technology, Konarskiego 18A, 44-100 Gliwice, Poland; 3 Department of Machine and Industrial Design, Faculty of Mechanical Engineering, VSB-Technical University of Ostrava, 17. Listopadu 2172/15, 708 00 Ostrava-Poruba, Czech Republic

**Keywords:** Mechanical rock mining, Cutting pick, A spraying of the cutting zone, Hydrodynamic rotation mechanism, Stresses, FEM simulation studies, Civil engineering, Mechanical engineering, Computational science

## Abstract

This article presents a proposal for a new solution of tangential-rotary picks intended for mining, especially hard rocks with roadheaders. The design of these picks is characterized by many innovative solutions aimed at significantly increasing their service life. The method to achieve this goal is to provide effective central spraying of the ring tip picks, crown picks, or similar types of picks while using the spray water to cause the picks to rotate as they contact the rock being mined. An important element of the work is to conduct extensive simulation studies using FEM to determine the stress distribution in the pick shank subjected to external load from cutting. The new design of the tangential-rotary pick is equipped with a reinforcing sleeve, in which a state of compressive stress is induced. This enables a significant improvement in the fatigue strength of the pick shanks. The conducted FEM comparative tests confirm the accuracy of the adopted design assumptions. They also determine the beneficial value of the tension nut torque, which tightens the reinforcing sleeve located on the pick shank.

## Introduction

Rock cutting in mining, tunneling, construction, and road works is most often carried out with the use of machines with cutting heads equipped with tangential-rotary (conical) picks. Conical picks became popular due to their great utility advantages. During the cutting of rocks, these picks are equipped with sintered carbide tips that are successively rotated around their longitudinal axis. As a result, the progressive abrasive and erosive wear of the picks has a uniform character, which determines their high mining efficiency. Thanks to the rotation of the picks, if they are not disturbed, long service life is maintained, energy consumption is limited, and the generation of rock dust, especially respirable dust that is dangerous to health, is minimized. However, in many cases during the mining operation, the picks do not rotate due to contamination of the pick holder sockets with stone dust consolidated with water and products of abrasive wear and corrosion of the pick shanks. The shanks are, thus, clamped in the pick holders. This results, among other issues, in asymmetric forms of pick tip wear^1–3^.

Mining machines are characterized by high work dynamics. This is especially true for cutting machines, as this process is a source of strong vibration excitations and dynamic loads. The size and nature of the load from the working process are important from the point of view of design machines to ensure their fatigue durability and reliability^4^. The picks of mining machines are subjected to highly variable loads, which are the reaction of the rock to the penetration of the tips. Therefore, these picks are one of the most strenuous elements of machines used in mining, road construction, or engineering construction. They determine, to a large extent, the efficiency of the mining machines. Ensuring the high durability of picks, especially those used for cutting hard surfaces and highly abrasive rocks, and a selection of cutting process parameters that are favorable in terms of wear, dynamic load, and fatigue life is, therefore, the subject of experimental and computer research conducted in various R&D centers over many years. These studies use modern research techniques, including the finite element method (FEM) and the discrete element method (DEM), as well as measurements at laboratory stations and in operational conditions. Theoretical and experimental research mainly concerns:

- the mechanics of rock cutting with conical picks^5^,
- the prediction of the load on conical picks and the energy consumption of mining, especially hard rocks^6–9^,
- the formation of stresses in the pick tip and its shaft^10–12^,
- the interaction of the pick with the pick holder^13^,
- the distribution of stresses in the pick holder with a pick placed within it^14^, and
- the wear mechanisms of the picks in the rock mining process^15^.

An essential factor accompanying the cutting, especially of hard and highly abrasive rocks, is to ensure adequate cooling of the picks by spraying the cutting zone with water. The sprinkling of water, in addition to the cooling of the picks, reduces the friction of the picks against the excavated rock massif and output, while wetting the produced dust particles facilitates their neutralization. To increase the effectiveness of combating the dust generated in the process of cutting with picks, special wetting agents are added to the water^16^. A very important function of water spraying is the early extinguishing of sparks that arise when cutting many types of rocks, which is particularly important in conditions of methane or other ignition hazards such as coal dust^17–19^.

There are many technical solutions for sprinkling, which have been described in detail, for example, in previous works^20,21^. In the case of tangential-rotary (conical) picks on the cutter head or cutter drum of mining machines, there are two main ways to direct the spray water stream: in front of the pick and behind the pick^22^. Attempts to use a central water supply through the tip of the pick are the most effective, especially because the high-pressure water jet supports for the mining process^21,23,24^ have not become popular due to numerous technical difficulties, including the clogging susceptibility of spray nozzles. In the commonly used button tips, there are also problems with creating sprinkling channels with a small diameter (about 1 mm) in tips with high hardness^25^ and ensuring their patency during operation.

The most commonly used, in practice today, is water spraying behind the pick, which is effective for facilitating the removal of mined material and reduces cutting resistance. The main disadvantage of this method, however, is that the cooling water stream falls onto the tip of the picks at the moment when they have the highest temperature, which results from the high work of friction converted into heat^26^. This causes a specific thermal shock of the tip material and promotes the initiation and development of the dangerous phenomenon of thermal-frictional fatigue, which strongly accelerates the degradation process of the conical picks^27^. A significant disadvantage of the currently used spraying systems is the high-water consumption and inefficient use. To reduce these unfavorable phenomena, attempts have been made for many years to use air–water aerosols for spraying, which are formed as a result of mixing water with compressed air in spraying nozzles. Such nozzles can be used directly on the cutter head of mining machines, which requires, however, water and compressed air to be supplied to these units through separate ducts^28^. An alternative solution, which is easier to implement, is the use of air-and-water curtains that act as external sprinklers; these are installed in the vicinity of the cutter head of the mining machines, for example, on the boom of the roadheader. The use of air-and-water spraying, among other benefits, leads to a significant reduction in water consumption in the spraying process of the mining zone. Water consumption per one spraying nozzle is, in this case, about 0.1–0.4 dm^3^/min^29^. Water consumption in such a situation is almost 2.5 times lower compared to conventional spraying systems^28^.

The rotation of the tangential-rotary (conical) picks during mining is carried out by using the component of the cutting resistance force, which creates the appropriate torque relative to the longitudinal axis of the picks. For this purpose, the picks are mounted onto pick holders that are set on the cutter head or cutter drum of the mining machines at the skew angle. However, this is associated with an increase in the general frictional resistance of the picks against the rock and a growth in the rate of abrasive and erosive wear; it is conducive to an increase in the temperature of the tips^30^. The basic disadvantage of this method of rotation of the picks is that the rotation is performed at the moments when they are most loaded, i.e., when the greatest resistances are opposing the rotation. This consumes an extensive amount of energy and increases wear on the bearing surfaces of the picks and pick holders. Hence, it is very inefficient.

The highly variable load of the tangential-rotary picks of an impact nature favors the formation of fatigue cracks of the picks, in particular, their shanks in the transition zone of the thrust collar into the gripping part of the pick body^31^. The highest bending moment and structural notch occur in the zone of transition radius. These factors significantly reduce the fatigue life of the pick shanks.

This article presents proposals for several structural changes to tangential-rotary picks, the aim of which is to improve the service life of the picks. Actions in this area are constantly updated because, especially when cutting hard surfaces and highly abrasive rocks, the intensity of pick destruction, to the extent that they need to be replaced, is very high. In such conditions, the consumption rate of picks may exceed 1 item/m^3^ of excavated rock^32^ and even reach 4 items/m^3^ (508 items/day)^33^. For example, when excavating a roadway or a tunnel with a cross-sectional area of 25 m^2^ with a depth of cut of 0.75 m, as many as 75 picks may be worn out in each mining cycle. This causes a vast increase in the costs of excavating due to the need to replace the entire set of picks located on the cutter heads or cutter drums of the mining machines during each shift. Rapid changes in the geometry and functional properties of the picks (caused by the high speed of their wear) also contribute to the increase in energy consumption of mining, the decrease in the efficiency of this process, the deterioration of the dynamic condition of the mining machine leading to its increased failure rate, and a significant deterioration in the working conditions and safety of mining crews.

The new solution of tangential-rotary picks, presented in this article, is the result of several decades of theoretical and experimental research and observation of the operation of mining machines in operating conditions. As part of the work on new solutions for picks for mining machines, numerical tests have been carried out to determine the state of effort for a new generation pick body equipped with a ring tip. The FEM studies included here, among others, compare the stress distribution in the critical cross-section of the conventional and modified pick shank.

## The concept of a spraying system integrated with the rotation of tangential-rotary picks

Central spraying during rock mining with tangential-rotating picks, in conjunction with the rotation of the picks around their axis, is performed automatically in each cutting cycle as a result of the use of a system that is activated by each pick separately at the moment of its contact with the excavated rock. The idea of individually powering the spraying nozzles assigned to individual picks was already known, for example, from the solutions used in Sandvik roadheaders. This eliminates the need to use sector distributors built inside the cutter head, characterized by high structural complexity and low durability and reliability (this applies, in particular, to seals operating in very difficult conditions)^34^. In the Sandvik solution, each pick holder has a valve that shuts off the water supply to the nozzle located in the pick holder. This valve opens under the pressure of the pick in contact with the mined rock^28^. Although the idea is similar, the solution presented here has a completely different effect. Spraying is central to an effective pick tip and occurs in two stages. The first stage begins when the tip of a given pick contacts the rock and is performed with heavily heated water due to the heat generated by the work of friction on the rock being mined. During the first stage of spraying, the pick rotates around the longitudinal axis by an elementary angle, which is successively summed up during each subsequent cutting cycle. The second stage of spraying, into which the system smoothly passes after the completion of the first stage, is carried out with a water stream with an increased degree of atomization. It lasts as long as the given pick remains in contact with the excavated rock and output. As soon as the pick stops contacting the rock, the flow of water is cut off.

Spraying in the first stage with heated water gently cools and lubricates the pick tip before it enters the main cutting stage. The use of highly heated water eliminates the thermal shock of the tip material and removes the heat generated during cutting to the outside of the cutter head or cutter drum of the mining machine, lowering its temperature. The elimination of thermal shocks of the tips, and the related limitation of repetitive cycles of changes in the temperature of the surface layer of the tip material, limit the possibility of nucleation and the development of thermal-friction fatigue of the picks. Surface cracks caused by thermal-friction fatigue lead to the gradual chipping of cemented carbide fragments, which leads to premature destruction of the picks^27^. Therefore, the limitation of sudden large temperature gradients of the tips is essential from the point of view of the service life of picks that are used for cutting rocks and other materials with similar mechanical properties.

**Figure 1:**
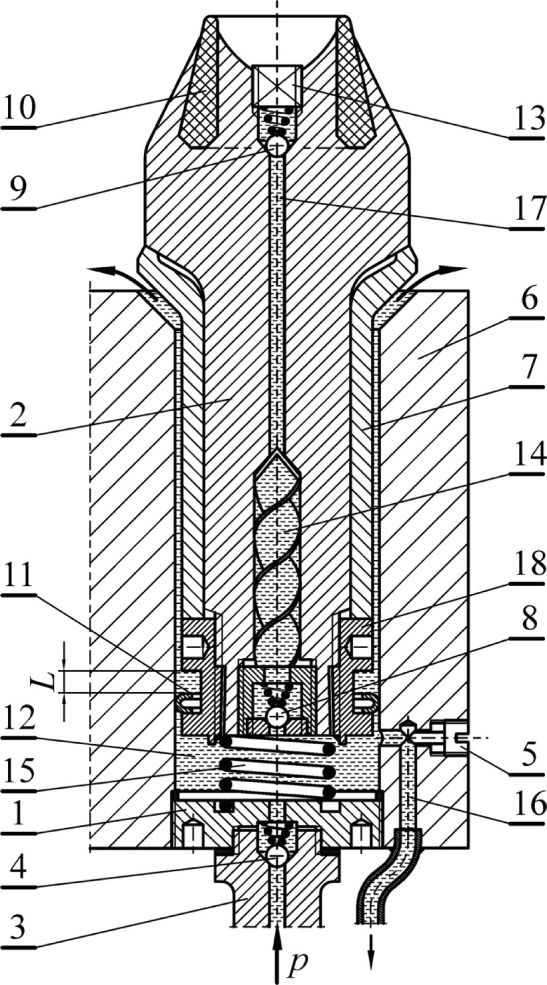
Solution of a two-stage hydrodynamic system of sprinkling and rotating picks that applies to picks with a ring-type tip (description in the text).

Figure 1 shows an exemplary solution of a two-stage hydrodynamic system of spraying and rotating picks that is applied to picks with a ring-type cutting tip. Sprinkling water is supplied from the central supply system individually to each pick holder through a stub pipe (3) equipped with a non-return valve (4) to the chamber (12) closed with a plug (1) and the face of the gripping part of the pick (2). During the standstill, and before the pick contacts the excavated rock, the water pressure in the chamber (12) is kept at a reduced level as a result of the operation of the overflow assembly with the flow resistance regulated by the choke (5). Simultaneously, a small part of the water, as a result of the slot flow, flows outside the pick holder through an appropriately selected gap between the socket in the pick holder (6) and the outer surface of the sleeve (7), reinforcing the pick shank. The slotted water outflow is used for flushing out dust impurities from the pick seating area in the pick holder. The outflowing water also washes away the dust impurities from the thrust surface of the pick holder, as shown by the arrows in Fig. 1. Check valves (8) and (9) are adjusted to remain closed during this time. Between valves (8) and (9), some of the spray water remaining from the previous cycle of the pick operation gradually heats up due to the energy received from the pick.

When the tip (10) of the pick contacts the rock being mined, the pick starts to move rapidly into the pick holder within the enlarged clearance *L* on the ring (11), protecting the pick from falling out of the pick holder. The longitudinal movement of the pick causes its shank, acting as a piston, to cause a sudden increase in water pressure in the chamber (12), closing the non-return valve (4) and blocking the outflow of water through the channel (16). The water hammer effect opens valves (8) and (9), and heated water gushes out of the nozzle (13), removing impurities from the nozzle outlet (i.e., the self-cleaning effect of the spray nozzles). The first stage of sprinkling begins, during which the heated water gently cools and lubricates the ring tip (10) before it begins the main cutting phase. This gently lowers the temperature of the pick tip after the previous cutting cycle without causing a thermal shock to the material from which the tip is made. This first stage of spraying lasts until the thrust collar of the pick contacts the front surface of the pick holder (6). During this spraying stage, the rapid flow of water through the reaction impeller (14) causes a reaction torque, which means that the pick rotates around its longitudinal axis by a small elementary angle^27^. The rotation of the pick is favored by the fact that it does not yet carry a significant load from the mined rock and, while in motion, it is lubricated with water from the slot flow, which overcomes a significantly reduced motion coefficient of friction.

The rotation of the pick, along with the longitudinal shift, lasts until the pick body contacts the front surface of the pick holder. Only then can the pick take over the full load from the cutting while closing the slotted water outflow. The closed overflow through the channel (16) causes the pressure in the chamber (12) to increase to the level of the supply pressure *p*. Hence, the check valves (8) and (9) remain open, and through the nozzle (13) a jet sprays the pick tip (10) and the cutting zone. This constitutes the second stage of spraying, during which the water flows through the immobilized impeller (14), undergoing a swirl around the axis of the stream. This facilitates the formation of an effective water mist, as the energy of the swirling motion of the spray jet is added to the energy of the longitudinal flow. The high degree of atomization of the spraying water effectively cools and lubricates the pick in its cutting phase and efficiently reduces the formation of dry rock dust, which is especially dangerous to respiration. This stage of spraying the cutting zone lasts until the pick losses contact with the excavated rock and the output material. Then the pressure of the water in the chamber (12), and the auxiliary action of the return spring (15), move the pick to the starting position. Check valves (8) and (9) close, and some of the water contained between them is warmed by receiving heat from the pick in preparation for a new cutting cycle. The slot flow and overflow channel discharges are opened. The pick in this position is completely ready to carry out the next two-stage spraying combined with rotation during the next cutting cycle.

The rotation of the pick, which always occurs in the same direction, depends on the direction of the impeller (14) winding and is successively summed up in subsequent cutting cycles. This ensures the uniform abrasive and erosive wear of the pick combined with a kind of “self-sharpening” effect in the course of mining. During the operation of the spraying system, combined with the rotation of the pick, there is the cooling of the pick from the inside and outside. This allows the maintenance of the full mechanical properties of the pick elements shaped in the technological process during its manufacturing.

Effective two-stage sprinkling using the hydrodynamic effect of water flow for the rotation of the picks makes it possible to resign from setting the picks on the working units of the mining machines at the tilt angle. Thanks to this, the cutting forces are reduced, and the intensity of the abrasive and erosive wear of the picks is lowered. This serves to increase the service life of the picks and enhances the energy efficiency of the rock mining process.

The use of picks with ring-type tips in the presented solution enables the convenient implementation of the most effective central spraying to the tips of the picks. Picks with ring tips are characterized by a long service life, which results from the fact that only a fragment of the tip is involved in the cutting process at a given moment. The remaining part of it can be freely cooled at this time and enters operation with a significantly reduced temperature after a full rotation of the pick around the longitudinal axis. Cutting picks with such a shaped tip are characterized by a significant reduction of cutting forces and mining energy consumption^35^. A particular advantage of the described solution is the very economical and effective water management system. Water spray is supplied only when it fulfills a specific utility function.

**Figure 2:**
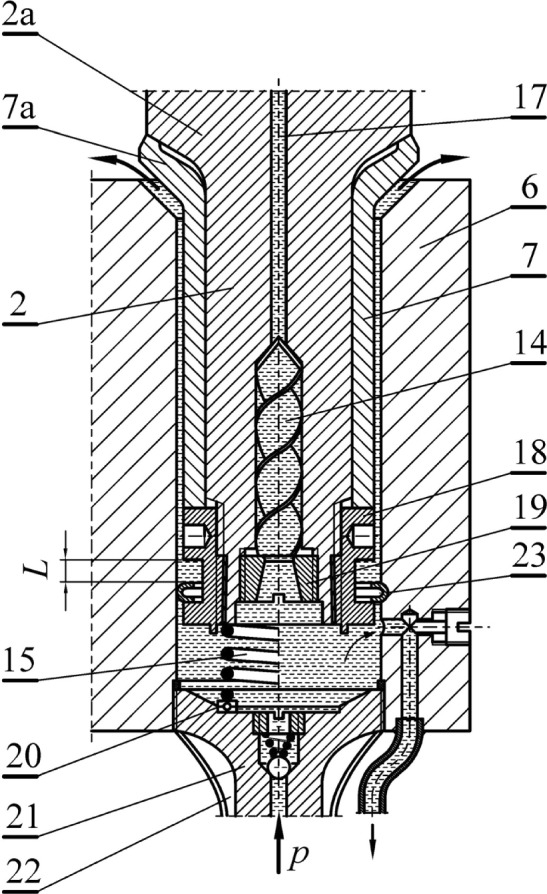
An alternative method of rotation using a hydrodynamic system (description in the text).

The layout concept allows for many modifications. With an increased feed water pressure *p*, it is possible to dispense with the return spring (15) (see the right-hand side of Fig. 2). The increased water pressure then provides a sufficient impulse to move the picks to the initial position, regardless of the position of the picks on the cutter head of the mining machine. Resignation of the return spring favors the rotation of the pick. With increased supply pressure, one should consider the increased slotted discharge, which may be limited by the reduction of the clearance between the pick shank and the inner surface of the socket in the pick holder. There may also be a need to protect the pick more permanently against being pushed out of the pick holder, for example, by making a recess (23) in the pick holder socket to seat the pick more firmly (see the right-hand side of Fig. 2). To reduce the effect of the return spring, it is possible to use a small roller bearing (20) (see the left-hand side of Fig. 2). It is advisable to use bearings made of corrosion-resistant material with ceramic rolling elements.

In this system, it is also possible to omit the check valve (8). In its place, a diffuser (19) can be used to accelerate the movement of water at the entrance to the impeller (14). This significantly facilitates the rotation of the knife in the first stage of sprinkling (Fig. 2).

A system without a check valve (8) may be desirable in the case of very intensive hard rock mining operations. In such situations, an increased release of heat may occur, leading to the evaporation of a part of the water inside the pick, especially in the central channel (17). The high heat of water vaporization serves to increase the heat removal from the pick. Due to the higher density of liquid water, the water vapor bubbles are forced into the chamber (12) under the pick shank during the rotation of the cutter head of the mining machine. Water at a lower temperature causes water vapor bubbles to condense there. The heat of water vapor condensation is transferred to the elements of the pick and the pick holder. The cooling ribs (22), made together with the cover (21), can help transfer this heat to the outside environment (Fig. 2). This heat transfer is achieved by convection and irradiation via the external surface of the ribs.

## Construction and technological modifications of tangential-rotary picks to increase the resistance of picks to fatigue breakage

### Characteristics of the solution

Fatigue damage to picks and, in particular, fatigue breakage of the shanks is a fairly frequent reason for the elimination of picks from the operation, which is strongly dependent on the conditions of use of tangential-rotating picks, including fluctuations in their load from cutting^12,21,27,36,37^. Shank fatigue fractures are particularly critical. While decisions to replace picks with new ones in the case of other forms of damage are made by the mining machine operator based on a visual assessment of the degree of wear, in the case of breaking shanks, both fatigue and ad hoc, picks “take themselves out of service” suddenly and without any clear earlier symptoms enabling pre-emptive action. The operation, even short-term operation of the cutter head/cutter drum of the mining machine with broken pick shanks, leads to serious damage to the pick holders, requiring troublesome repair operations in factory conditions. Therefore, the fatigue life of the tangential-rotary pick shanks is of great importance from the point of view of reliability.

The Department of Mining Mechanization and Robotisation at the Silesian University of Technology developed many structural and technological modifications^36^ aimed at improving the fatigue life of pick shanks. They mainly consist of the use of various strengthening treatments, including hardening through burnishing performed by pressing^38^, the dynamic method^39^, and combined methods^40^.

Design solutions that improve the fatigue resistance of pick shanks include, among others, the use of auxiliary reinforcing elements, for example, in the form of a special sleeve^27^. Figure 1 illustrates the use of the reinforcing sleeve (7) mounted on the pick shank (2). A thin-walled sleeve (7) made of high-fatigue steel is positioned with slight pressure on the pick shank (2), after which it is subjected to a state of significant compressive stress, obtained by tightening the tightening nut (18) firmly, preferably equipped with a fine thread. During assembly, it is helpful to heat the pick shank and cool the reinforcing sleeve to obtain a higher level of compressive stress in the sleeve after the temperatures are equalized. After final assembly, the tightening nut is secured against any loosening, for example, by a spot weld or a small tack weld.

**Figure 3:**
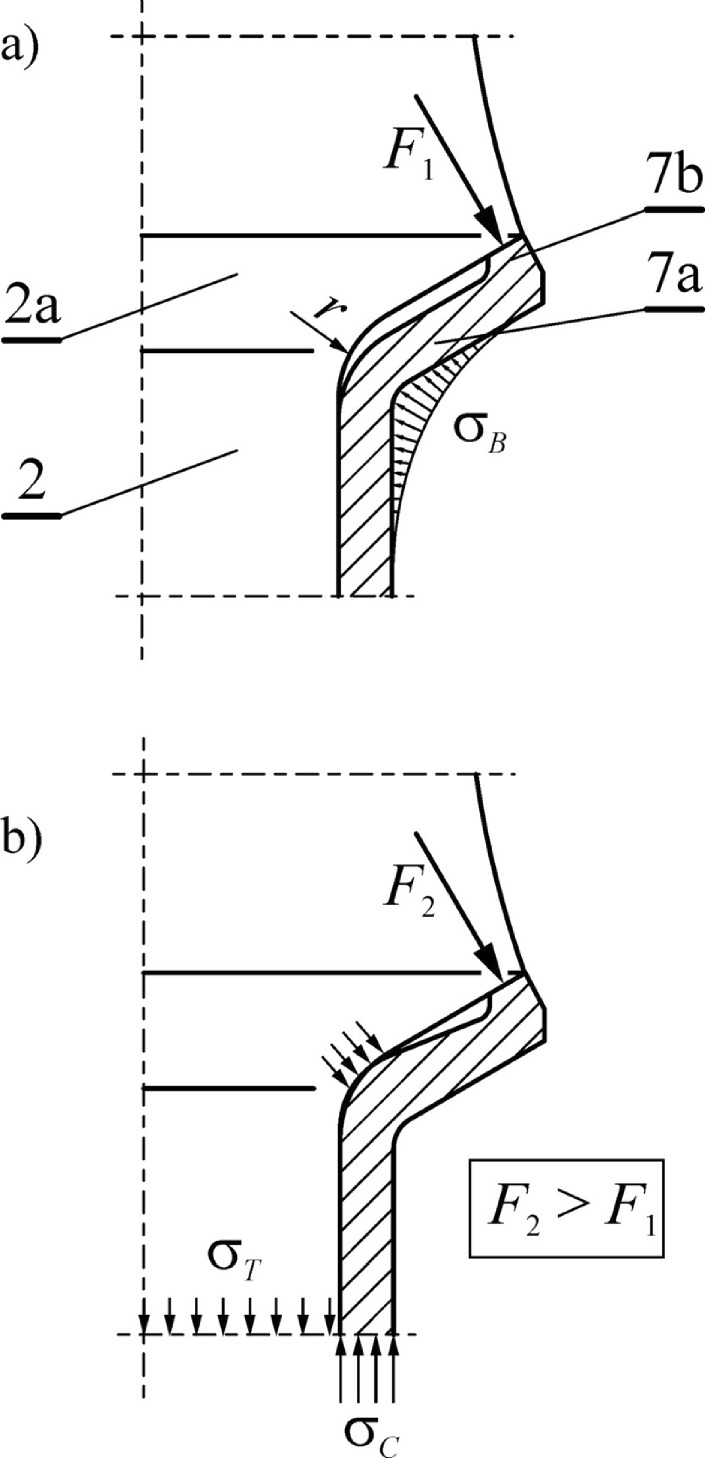
State of stress in the reinforcing sleeve’s cup-shaped collar: (**a**) the effect of the bending moment of the collar with a shaped protrusion, and (**b**) the state of stress after the inner surface of the sleeve’s cup-shaped collar contacts the thrust collar of the pick shank.

The reinforcing sleeve, being in a state of initial tension, has a very significant impact on the state of stress in the critical cross-section of the pick shank, where fatigue cracks most likely occur. It is particularly advantageous to equip the reinforcing sleeve with a flange, preferably in the shape of a cup (see Fig. 2). When a protrusion (7b) is formed on the outer rim of the socket flange (7a) then, as a result of tightening the tightening nut (18), an additional state of stress is caused on the outer side in the form of compression due to the action of the bending moment of the force *F*_1_. This is schematically illustrated in Fig. 3a. The bending stresses *σ*_*B*_ of the reinforcing sleeve flange, as a result of superposition with the compression stresses *σ*_*C*_ caused by the tightening of the tightening nut, change the state of the stresses in the critical cross-section of the pick particularly favorably. The oscillatory cycle of stress change, occurring in the pick shaft without a sleeve, is adapted into a cycle pulsating largely on the compression side. This significantly increases the fatigue strength of the composite pick shank-reinforcing sleeve system. Additional tensile stresses *σ*_*T*_ in the pick shank, resulting from the initial tension of the sleeve, are small due to the cross-section of the pick shank being many times larger than that of the thin-walled reinforcing sleeve. In addition, these stresses already occur in the zone with reduced stresses from bending the pick. The protrusion (7b) is selected in such a way that, at the final degree of tightening the tightening nut (18), the inner surface of the cup collar of the reinforcing sleeve contacts the thrust collar (2a) of the pick shank (Fig. 3b). The mechanism of shaping the state of stress in the pick shank, due to the use of a reinforcing sleeve, has been previously described in ref.^27^.

During the assembly of the tightening nut (18), the moment of contact of the reinforcing sleeve flange with the thrust collar of the pick shank arises in the form of a sudden increase in the tightening resistance of the tightening nut, which is caused by a large increase in system stiffness. The elasticity of the reinforcing sleeve flange means that the favorable state of stress in the critical cross-section of the pick shank is maintained, even at the temperature difference of the elements during operation. The cup shape of the collar is conducive to the use of an increased radius of transition *r* of the pick shank thrust collar into its gripping part (Fig. 3a). This serves to reduce the notch in this zone, which further increases the resistance to fatigue breakage of the picks.

The conical shaping of the front surface of the pick holder (6) makes it possible to increase the counter surface without increasing the outer diameter of the working part of the pick body. In addition, it facilitates the removal of dust pollution in this zone (see Fig. 1). The outflowing water washes away smaller impurities, and larger ones moistened with water are more easily thrown out by the centrifugal force during the rotation of the cutter head of the mining machine. Increased resistance to the rotary movement of the picks (around their longitudinal axes under load) protects against undesirable slips that cause abrasive and erosive wear of the resistance surfaces of the picks, especially the pick holders. For the desired rotation of the picks around the longitudinal axis, they are not important; this is because the rotation of the picks occurs with a small load on the picks, as described in section “The concept of a spraying system integrated with the rotation of tangential-rotary picks”.

### A modified design form of a tangential-rotary pick

**Figure 4:**
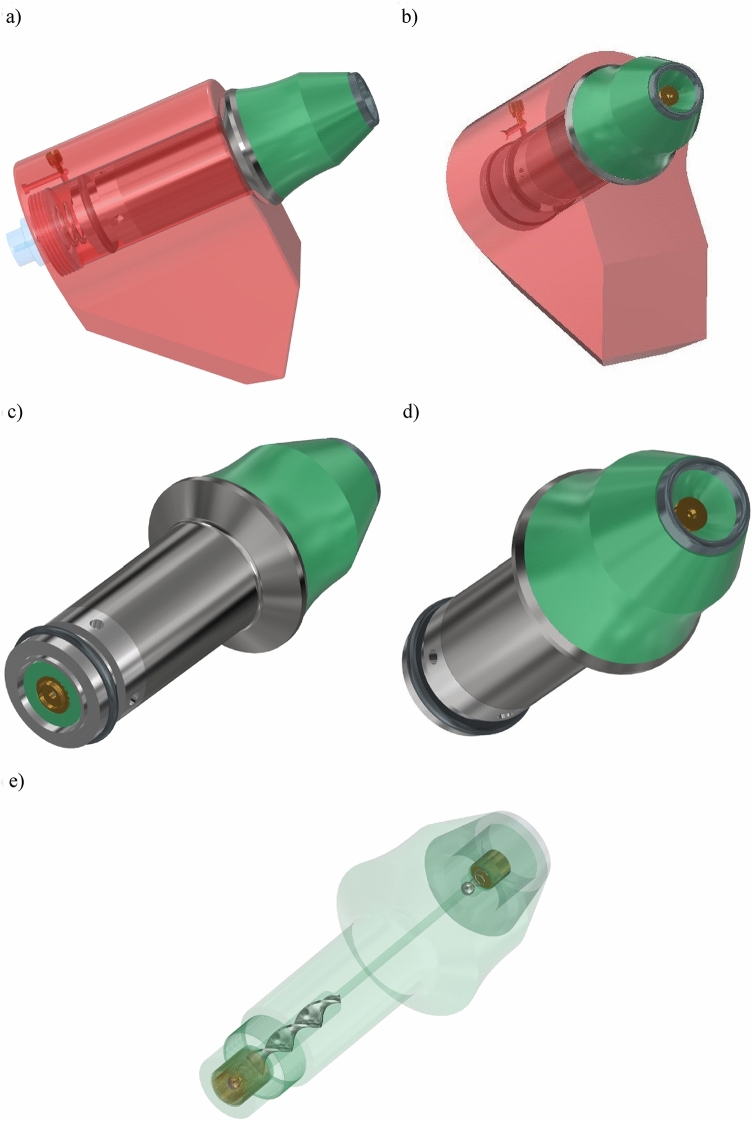
3D model of a pick with a ring-type tip equipped with a hydrodynamic sprinkling and rotation system: (**a**, **b**) view of the pick mounted in a pick holder with a modified design, (**c**, **d**) view of the complete pick with a reinforced sleeve, check valve, and spraying nozzle, and (**e**) view of the impeller mounted inside the pick shank.

Based on the description in section “Characteristics of the solution” of the concept of a tangential-rotary pick that is equipped with an impeller and a reinforcing sleeve, a design of a structural solution is developed. The CAD model of a pick with a ring tip inserted in a pick holder, adapted for hydrodynamic central spraying and pick rotation, is shown in Fig. 4a and b. In turn, Fig. 4c and d display the pick equipped with a reinforcing sleeve and a tightening nut. The pick has elements of the hydrodynamic spraying system, including a spray nozzle screwed into a socket made in the recess of the working part of the pick body, inside the ring tip (Fig. 4d) and a check valve holder located at the rear end of the gripping portion of the pick body (Fig. 4c). In the central channel of the pick shank, there is an impeller responsible for the hydrodynamic rotation of the pick (Fig. 4e). This drawing also shows a set of check valves built into the pick body in its rear part and under the spray nozzle. In the developed form of the pick, the ball check valves were used.

**Figure 5:**
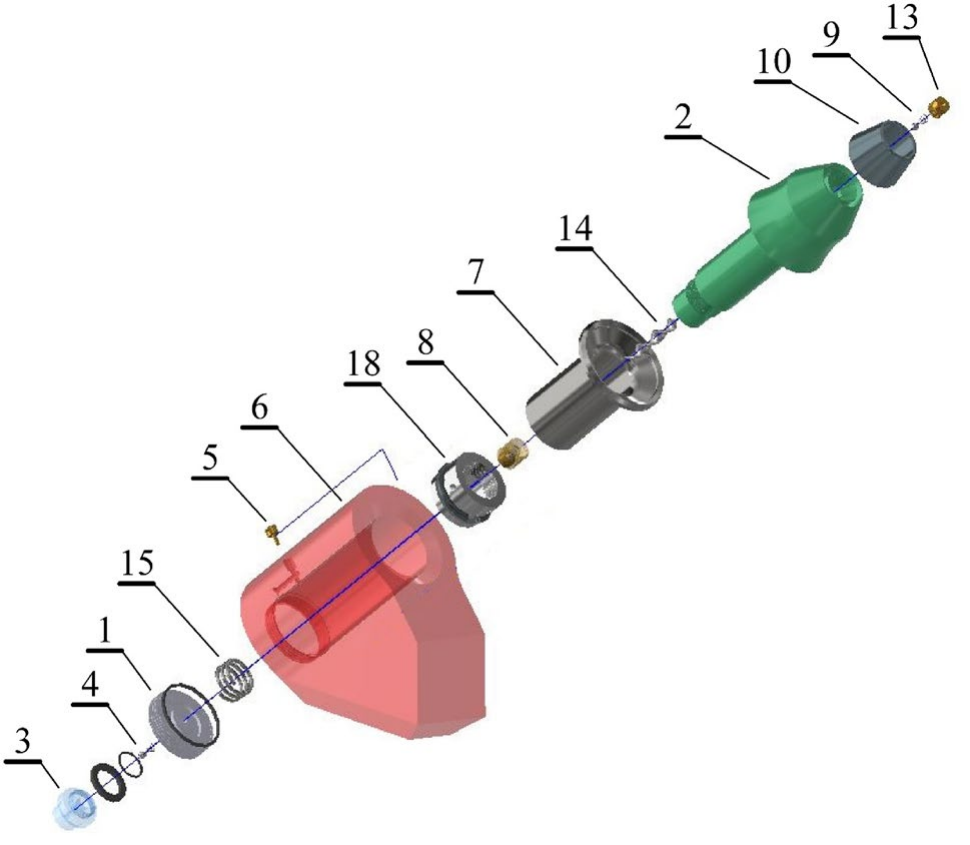
Expanded view of a pick equipped with a hydrodynamic sprinkling system and its rotation (markings given in the text).

The components of the assembly include the tangential-rotary pick assembly with a ring tip – the pick holder in the developed version is shown in Fig. 5 (the markings are analogous to Fig. 1). The complete pick assembly consists of a monolithic steel shank (2) with a diameter of *ϕ*34 mm with a hollow, properly shaped central channel and a threaded end (a fine thread M27 × 0.75 mm was used). Accounting for the diameter of the pick shank thrust collar and its diameter, a transition radius of *r* = 7.5 mm was used (according to generally accepted rules of diameter gradation, it should be greater than 6 mm). In its front part (working part), there is a ring tip (10) made of sintered carbides, a check valve (9), and a spray nozzle (13). In its rear part (i.e., a gripping part), there is an impeller (14) and a check valve (8). A reinforcing sleeve (7) with a diameter of *ϕ*38/34 mm is placed onto the gripping part of the pick body, which is its core (typical tangential-rotating picks used for mining hard rocks have a shank with a nominal diameter of *ϕ*38 mm). This sleeve is pressed against the thrust collar of the pick shank using a tightening nut (18). The latter is equipped with a spring ring embedded in a groove, protecting the pick against falling out of the pick holder. Simultaneously, this ring ensures the possibility of axial movement of the pick in the pick holder within the assumed clearance. The pick holder (6), in which the pick is mounted, is a modified version of the holder with a classic design with a socket with a nominal diameter of *ϕ*38 mm for tangential-rotary picks. This modification consists of closing the lower end of the socket, in which the pick is mounted, with a plug (1). A stub pipe (3) is screwed into this plug, supplying water to the spraying system from the sidewall of the cutter head of the mining machine. Inside the plug (1), there is a non-return valve (4) and a return spring (15). In the body of the pick holder, there are channels of the overflow assembly that is equipped with a choke (5).

### Computer studies of the state of stress in the pick shank equipped with a reinforcing sleeve

**Figure 6:**
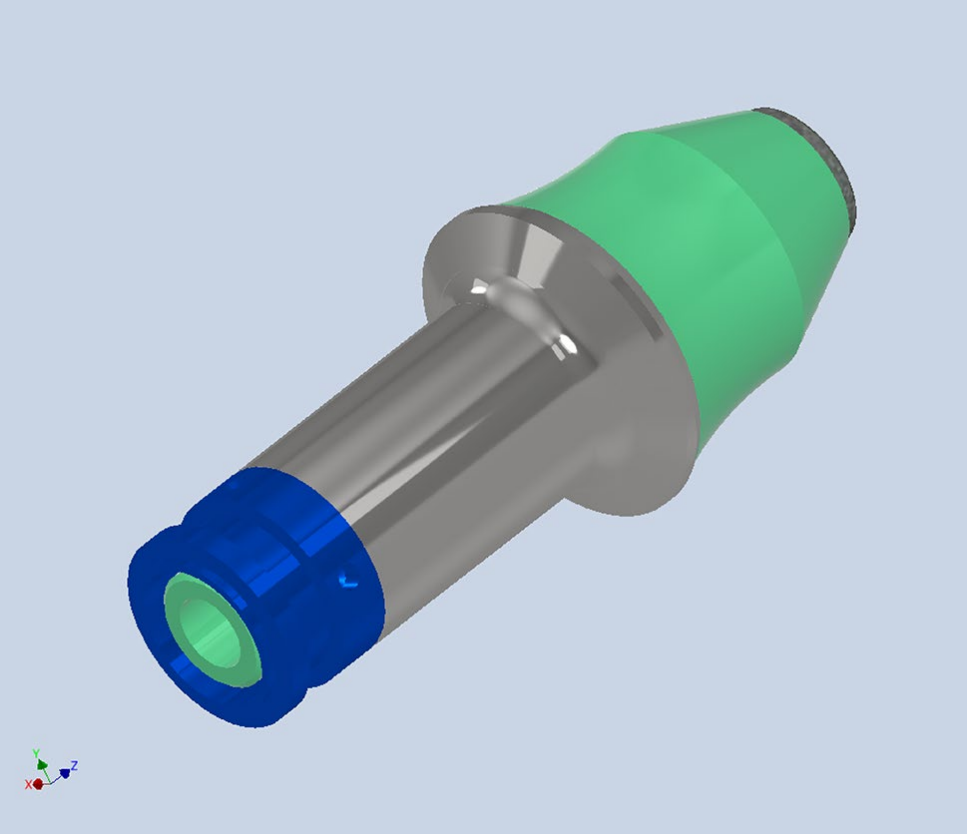
Simplified CAD model of a tangential-rotary pick equipped with a reinforcing sleeve for the FEM stress state simulation.

To determine the effect of the preload of the reinforcing sleeve on the state of stress in the pick shank, extensive computer studies using FEM are carried out. These tests are performed in the Autodesk Inventor Nastran 2022 software. A pick model developed by Autodesk Inventor is used for this purpose (Fig. 6). After simplification, this model consists of the following bodies: pick shank, reinforcing sleeve, tightening nut, and ring tip. To study the effect of the use of a reinforcing sleeve on the stresses in the pick shank, a CAD model of a pick of identical shape and dimensions but equipped with a steel monolithic shank with a diameter of *ϕ*38 mm is built. This diameter corresponds to the constructions of tangential-rotating picks commonly used for mining hard rocks.

#### Pick model with a ring tip

**Figure 7:**
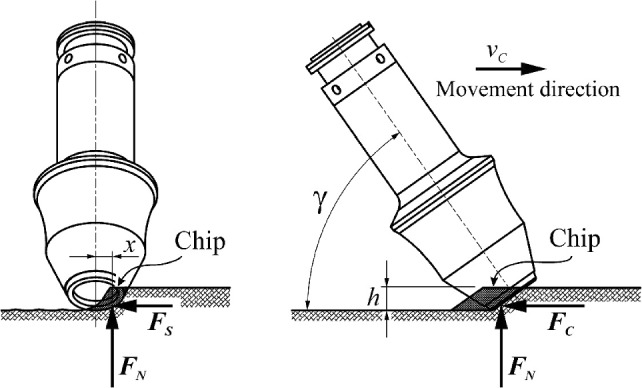
Scheme for setting a pick with a ring-type tip onto the surface of the rock being mined and the forces acting on the tip during cutting.

The starting point for determining the stresses in the pick shank using the finite element method is to determine its load state and the method of support in the pick holder. The process of rock mining with the depth of cut *h*, while moving the pick at the cutting speed *v*_*C*_, is considered (Fig. 7). The pick is set to the surface of the cut rock at an attack angle *γ*.

**Figure 8:**
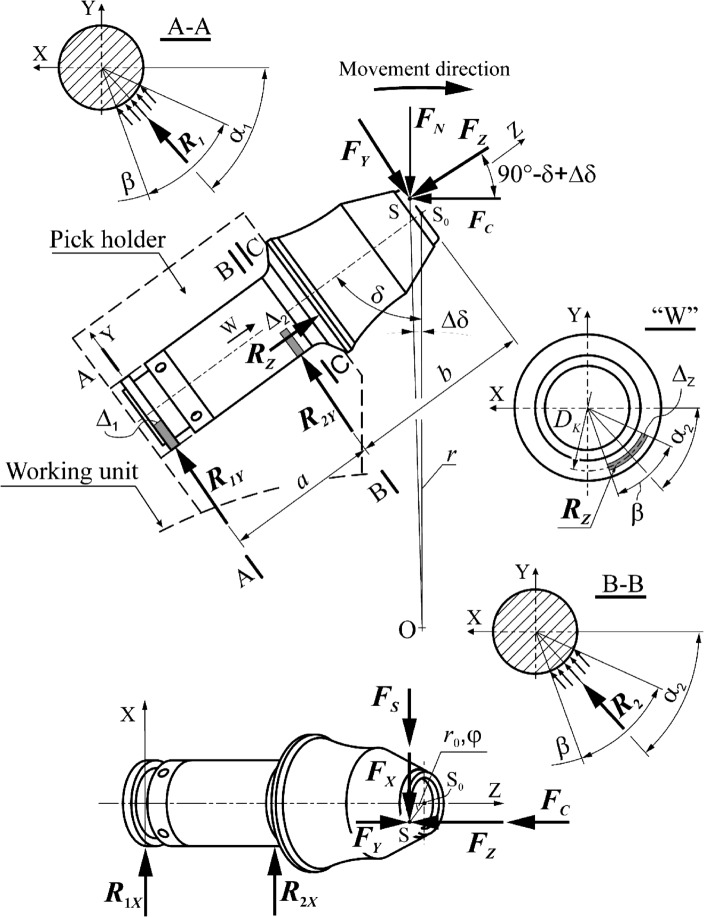
Method of modeling of the support of the pick shank in the pick holder for the needs of the FEM analysis.

The tangential-rotary pick is subjected to cutting resistance, which is commonly represented by three components: cutting force *F*_*C*_, normal force *F*_*N*_, and side force *F*_*S*_^41^. For picks with a conventional tip shape (a conical tip), the resultant rock reaction acting on the pick is hooked at the tip. In the case of a ring tip, point S_0_ is a hypothetical point of intersection of the longitudinal axis of the pick with a plane perpendicular to it, i.e., tangential to the surface of the tip (Fig. 8). Hence, the resultant rock-cutting resistance force is applied at point S on the contact surface of the pick tip with the rock being mined. Depending on the cutting pitch, this point is displaced by the length *x* from the plane of symmetry of the pick (i.e., the plane perpendicular to the surface of the rock being mined, passing through the axis of the pick)—see Fig. 7. In fact, the external load of the pick is distributed over the contact surface of its tip with the cut rock. Therefore, when modeling the external load of the tested pick, the components of the cutting resistance are distributed evenly on the ring surface, with the width resulting from the shape of the pick edge limited by the angle. The location of the central point of this surface (point S) is determined by coordinates in the polar coordinates system (*r*_0_, *φ*)—see Fig. 8.

The load on the pick during mining is balanced by the reactions in the pick holder socket. Due to the presence of clearances in the setting of the pick in the pick holder (in typical solutions, this is about 0.2–0.5 mm), the pick may contact the pick holder in various ways^5^. In this paper, the case in which this contact occurs at the opposite ends of the gripping part of the pick body and on the periphery of its thrust collar is considered. Thus, the impact of the pick holder on the pick is presented in the form of three reactions (at this stage treated as concentrated forces), i.e., radial forces *R*_1_ and *R*_2_ and the axial force *R*_*Z*_ applied at the point located on the contact surface of the thrust collar with the front surface of the pick holder. Resultant reactions in the direction perpendicular to the axis of the pick are applied at the opposite ends of the socket in the pick holder, displaced from the point S_0_ by *b* (reaction *R*_2_) and *a* + *b* (reaction *R*_*1*_), respectively. The resultant reactions at these points deviated from the X-axis of the coordinate system associated with the pick, as seen in Fig. 8, by the angle *α*_1_ and *α*_2_, respectively. These angles determine the location of the contact zones of the pick body with the surface of the socket in the pick holder. The value of the angle *α*_2_ also determines the location of the foothold of the *R*_*Z*_ reaction. It is assumed that the contact of the pick thrust collar with the front surface of the pick holder occurs near the place where the pick shank is in contact with the surface of the socket in the pick holder. Determining the values of angles *α*_1_ and *α*_2_ requires the solving of the following system of equilibrium equations (Fig. 8):

1 $$\left\{\begin{array}{c}\begin{array}{c}\begin{array}{c}{R}_{1X}+{R}_{2X}-{F}_{X}=0\\ {R}_{Z}-{F}_{Z}=0\\ -{R}_{2X}\cdot a-{0.5R}_{Z}\cdot {D}_{K}\cdot cos{\alpha }_{2}+{F}_{X}\cdot \left(a+b\right)=0\end{array}\\ {R}_{1Y}+{R}_{2Y}-{F}_{Y}=0\end{array}\\ {R}_{2Y}\cdot a+{0.5R}_{Z}\cdot {D}_{K}\cdot sin{\alpha }_{2}-{F}_{Y}\cdot \left(a+b\right)=0\end{array}\right.$$

and

2 $$\begin{gathered} {\text{tan}}\alpha_{1} = \frac{{R_{1Y} }}{{R_{1X} }} \hfill \\ {\text{tan}}\alpha_{2} = \frac{{R_{2Y} }}{{R_{2X} }} \hfill \\ \end{gathered}$$

The components of the pick’s load during the cutting can be reduced to an XYZ reference system, which is expressed as follows:

3 $$\left\{\begin{array}{c}{F}_{X}={F}_{S}\\ {F}_{Y}={F}_{N}\cdot \text{sin}\left(\delta -\Delta \delta \right)-{F}_{C}\cdot \text{cos}\left(\delta -\Delta \delta \right)\\ {F}_{Z}={F}_{N}\cdot \text{cos}\left(\delta -\Delta \delta \right)+{F}_{C}\cdot \text{sin}\left(\delta -\Delta \delta \right)\end{array}\right.$$

where *D*_*K*_ represents the diameter of the circle on the surface of the pick’s thrust collar on which the *R*_*Z*_ reaction application point is located, *δ* signifies the angle of setting the pick on the cutter head or cutter drum of the mining machine between the longitudinal axis of the pick and the straight line passing through the axis of rotation of the cutter head and the point S_0_. In addition, Δ*δ* is an angular shift of the foothold of the resultant cutting force (point S) to point S_0_, and *F*_*C*_, *F*_*N*_, and *F*_*S*_ are the cutting force, normal force, and side force, respectively.

It is assumed that the reactions in the positions where the pick is supported in the pick holder are in the form of a load distributed over a certain surface, which results from the contact range of the cooperating elements. These surfaces (marked in gray in Fig. 8) are defined by the angle *β* and the parameters *Δ*_1_, *Δ*_2_, and *Δ*_*Z*_. The friction of the pick in the pick holder is neglected.

**Figure 9:**
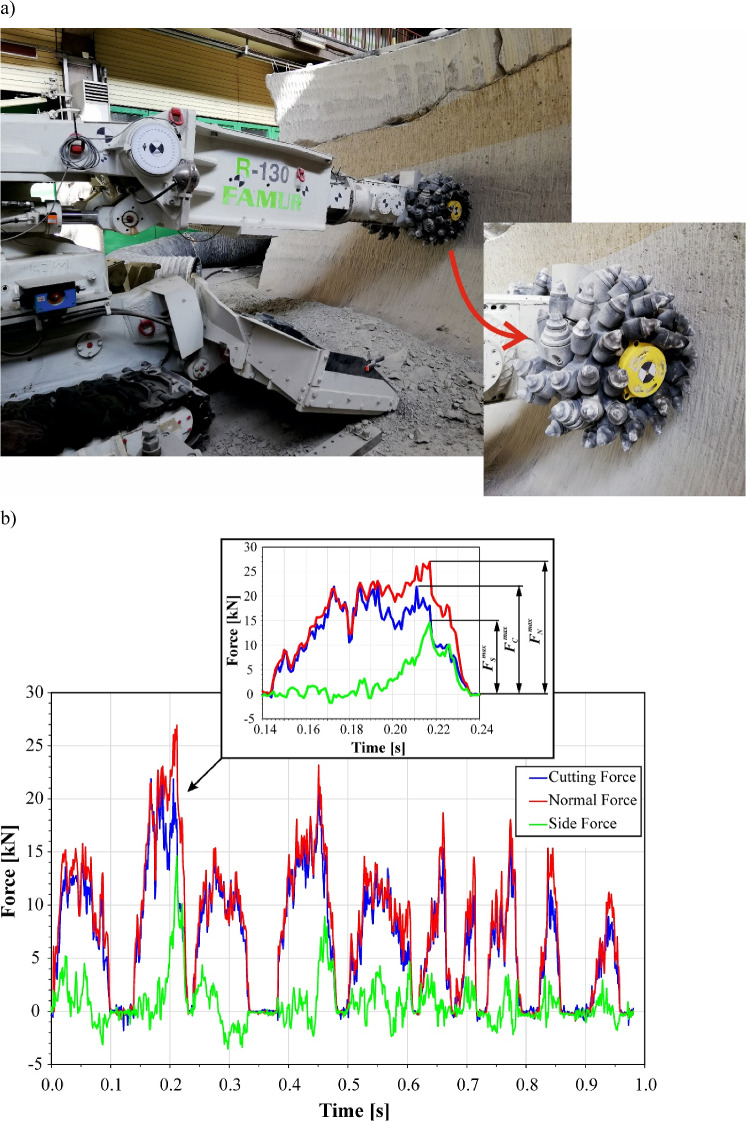
(**a**) Measuring stand with the FAMUR R-130 roadheader and (**b**) an exemplary course of the actual load on the pick during cutting of rock with compressive strength UCS = 50 MPa (in **a**, the arrow shows the pick holder on the cutting head equipped with a three-axis force sensor, the yellow element is the picks’ load recorder).

To determine the stresses in the shank of the pick during the working process, the actual waveforms of the load components of the tangential-rotating pick during mining of the cement-sand block with the FAMUR R-130 roadheader at the test stand are used (Fig. 9a)^42^. During the cutting of concrete with uniaxial compressive strength UCS = 50 MPa, the time courses of the forces relating to cutting, normal, and side in successive rotations of the cutter heads are recorded (Fig. 9b). The forces acting on the pick were measured using a triaxial piezoelectric force sensor KISTLER 9077C. This sensor is built into a pick holder with a specially adapted design, attached to the side surface of the cutter head of a roadheader. The time characteristics of the cutting load components were recorded using a data recorder built into the cutter head hub. This system enabled the recording and transmission over the WiFi network of measurement signals from three force sensors (nine channels) with a frequency of 1 kHz. The technical and metrological data of the applied force sensors are presented in Table 1. As can be observed, the load on the pick is characterized by high variability, which is typical when mining rocks and other brittle materials. Subsequent cutting cycles correspond to an intensive increase in the load on the pick from zero (from the moment the pick contacts the excavated rock), load oscillations resulting from the chipping of successive grains of the excavated material, and then pick unloading after it leaves contact with the rock being mined. The duration of such cycles results from the cutting speed *v*_*C*_ and the length of the path on which this process is performed. In the example shown, these cycle times varied, which resulted, among others, from the variable angular velocity of the cutter heads of the tested roadheader, driven by an electric motor powered by a converter system. They did not exceed 0.1 s in this work.

**Table 1:** Basic technical and metrological data of the KISTLER 9077C force sensor^44^.

Parameter		Unit	Value
Range	*F*_*X*_, *F*_*Y*_	kN	− 75… + 75
	*F*_*Z*_	kN	− 150… + 150
	*F*_*Z*_	kN	0…500
Overload	*F*_*X*_, *F*_*Y*_	kN	− 90… + 90
	*F*_*Z*_	kN	− 180… + 180
Calibrated range	*F*_*X*_	kN	0…75
	*F*_*Y*_	kN	0…75
	*F*_*Z*_	kN	0…150
	*F*_*Z*_	kN	0…500
Permissible moment load	*M*_*X*_, *M*_*Y*_	Nm	–2,040… + 2,040
	*M*_*Z*_	Nm	–2,040… + 2,040
Threshold		N	< 0.01
Sensitivity	*F*_*X*_, *F*_*Y*_	pC/N	~ − 4.2
	*F*_*Z*_	pC/N	~ 2.0
Linearity incl. hysteresis, each axis		%FSO	≤ ± 0.25
Crosstalk	*F*_*Z*_ → *F*_*X*_, *F*_*Y*_	%	≤ ± 0.5
	*F*_*X*_ ↔ *F*_*Y*_	%	≤ ± 1.5
	*F*_*X*_, *F*_*Y*_ → Fz	%	≤ ± 1.5
Rigidity	*C*_*X*_, *C*_*Y*_	N/μm	~ 8,400
	*C*_*Z*_	N/μm	~ 26,000
Operating temperature range		°C	− 40… + 120
Insulation resistance at 20 °C		Ω	> 10^13^
Capacitance, each channel		pF	1000
Weight		g	1040
Degree of protection according to EN60529			IP65

In the presented studies, the state of stress of the pick bar loaded with static forces of the maximum value in the selected mining cycle is analyzed. The course of the forces on the picks is a zero-based cycle with a maximum value ($\text{denoted by} {F}_{C}^{max}$, ${F}_{N}^{max}$, $\text{and} {F}_{S}^{max}$)—see Fig. 9b. A fragment of the course of this load in the considered mining cycle is shown (enlarged in the window at the upper part of Fig. 9b). In the FEM analysis, the pick model is loaded with the static forces *F*_*X*_, *F*_*Y*_, and *F*_*Z*_ that are determined from formulas (3), then substituting the maximum cutting, normal, and side forces for the components of the load on the pick means that: ${F}_{C}={F}_{C}^{max}$=22.2 kN, ${F}_{N}={F}_{N}^{max}$ = 26.5 kN, and ${F}_{S}={F}_{S}^{max}$=14.6 kN. The tests covered a pick with a conventional (monolithic) shank with a diameter of 38 mm and a pick with the proposed design, equipped with a reinforcing sleeve preloaded with a tightening nut, as described in section “The concept of a spraying system integrated with the rotation of tangential-rotary picks”.

The 3D model of the pick (Fig. 6) is meshed. The size of the mesh elements is assumed to be 2 mm, except for the following:

- the reinforcing sleeve (i.e., the thin-walled element), for which the size of the elements is 0.5 mm,
- the thrust collar and the transition radius of the pick shaft, where the size of the elements is 1 mm.

The individual parts of the pick are modeled using volumetric elements in the form of tetrahedral solid elements of the parabolic type. The pick model equipped with a reinforcing sleeve consists of 1,229,600 nodes and 789,681 elements. The connection of the tip with the working part of the pick body, and the threaded connection of the tightening nut with the pick shank, are modeled using bonded surface contacts. The contact of the reinforcing sleeve flange with the pick thrust collar is modeled in the form of surface contact of the sliding-no separation type. This ensures that the flange of the reinforcing sleeve can be moved relative to the thrust collar of the pick shank. Similar is the contact of the reinforcing sleeve with the pick shank on the cylindrical surface. Friction on the contact surfaces of the cooperating elements is omitted. It is assumed that the pick shank and the reinforcing sleeve are made of structural alloy steel for thermal improvement 35HGS (PN-89/H-84030/04), i.e., the material commonly used for tangential-rotary pick shanks^43^. The tightening nut is made of steel C45E/1.0503 (EN 10083-2). Basic material data for the set-up is presented in Table 2.

**Table 2:** Material properties of the elements of the new design of the tangential-rotary pick adopted for FEM tests.

Element	Material properties				
	Steel grade	Density	Elastic modulus	Tensile strength/endurance limit	Poisson’s ratio
		[kg/m^3^]	[GPa]	[MPa]	[–]
Pick shank	35HGS	7850	210	1650/750	0.3
Reinforcing sleeve	35HGS			1650/750	
Nut	C45E			650/290	

**Figure 10:**
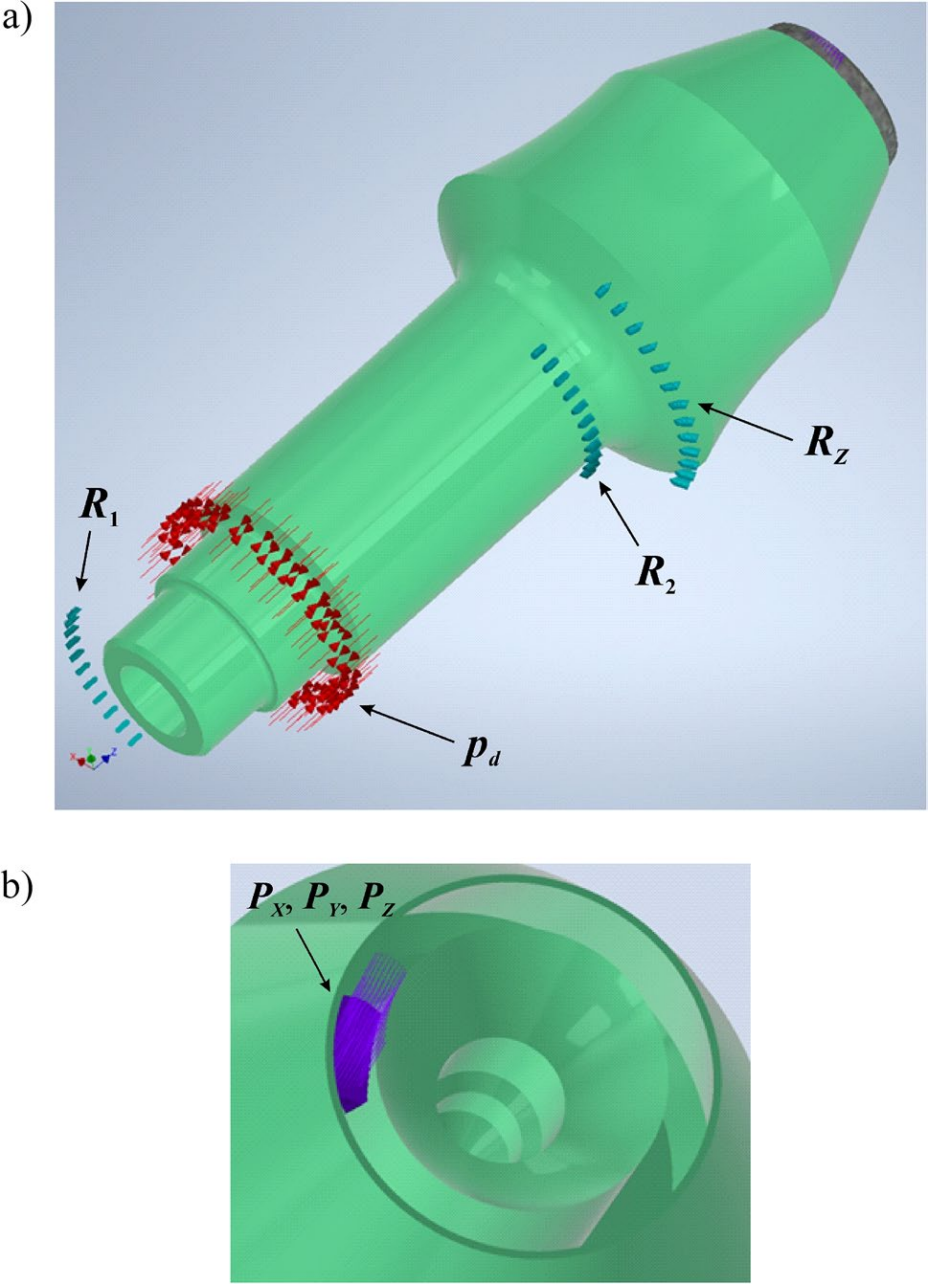
Method of modeling of (**a**) the constraints in the positions where the pick shank is supported in the pick holder (arrows in light blue) and the preload of the reinforcing sleeve (arrows in red), and (**b**) components of the load on the pick during cutting (arrows in purple)—for clarity, the reinforcing sleeve, tightening nut, and ring tip are not shown in the drawings.

The support of the pick shank in the pick holder is modeled in the form of evenly distributed reaction forces, hooked in the nodes of the grid of the designated areas of the outer surface of the reinforcing sleeve and the tightening nut (Fig. 10a—blue arrows). It is assumed that the pick contacts the pick holder on contact surfaces limited by the angle *β* = 90° and width *Δ*_1_= *Δ*_2_ = *Δ*_*Z*_ = 2 mm (Fig. 8). An even pressure distribution is presumed between the gripping part of the pick body and the socket in the pick holder, as well as the thrust collar and the front surface of the pick holder.

To examine the effect of the preload of the reinforcing sleeve, different values of the tightening torque of the *M*_*d*_ tightening nut are considered. The analysis of the thread load capacity for the connection of the tightening nut with the pick shaft shows that the limiting value of the tightening torque *M*_*d*_ is the permissible surface pressure in the threaded connection. For the assumed material properties of the tightening nut and pick shaft, as well as the geometry of the threaded connection, the value of the reinforcing sleeve preload should not exceed 88 kN. The tightening torque of the tightening nut should, therefore, be less than 195 Nm. For this reason, the values of the nut tightening torque ranging from 5 to 175 Nm are adopted for the tests (Table 3). The individual values of this moment correspond to the values of the initial tension force of the reinforcing sleeve and the pick shank, *Q*_*d*_, is determined from the following formula (without the friction of the nut against the reinforcing sleeve):

4 $$Q_{d} = \frac{{M_{d} }}{{0.5d_{S} \cdot \tan \left( {\gamma + \rho } \right)}} \cdot 10^{ - 3}$$

where *Q*_*d*_ signifies the initial tension force (in units kN), *M*_*d*_ corresponds to the nut tightening torque (Nm), *d*_*S*_ is thethread diameter (m), *γ* is the thread helix angle (deg), and *ρ* is the angle of friction in a threaded connection (deg).

**Table 3:** Values of the tension force of the reinforcing sleeve and the pressure constituting the FEM model load as a function of the tightening torque of the tightening nut.

*M*_*d*_	*Q*_*d*_	*p*_*d*_
[Nm]	[kN]	[MPa]
5	2.3	10.2
10	4.5	20.4
15	6.8	30.6
20	9.0	40.8
25	11.3	51.0
50	22.6	102.0
75	34.0	153.0
100	45.0	204.0
125	56.5	250.0
150	68.0	300.0
175	79.0	351.0

In the developed FEM model, the preload force is applied in the form of a pressure *p*_*d*_ with uniform distribution on the contact surfaces of the tightening nut with the reinforcing sleeve (red arrows in Fig. 10a). The values of these pressures are calculated as the ratio of the preload force to the face area of the reinforcing sleeve, i.e.:

5 $$p_{d} = \frac{{4Q_{d} }}{{{\uppi }\left( {D^{2} - d^{2} } \right)}} \cdot 10^{ - 3} { }$$

where *p*_*d*_ is the pressure on the contact surfaces of the reinforcing sleeve and the tightening nut (MPa), and *Q*_*d*_ is the preload force of the reinforcing sleeve (kN); additionally, *D* and *d* are the external and internal diameter of the reinforcing sleeve, respectively (m).

Figure 10b shows the method of applying the external load to the pick (for clarity, the ring tip has been removed from view). The components of the cutting resistance, reduced to the XYZ coordinate system, uniformly load the ring tip on the surface of its contact with the mined rock (lines in purple). It is assumed that this surface is limited by the central angle *Δα* = 30°, with the angle *φ* = 7.5° (Fig. 8).

**Figure 11:**
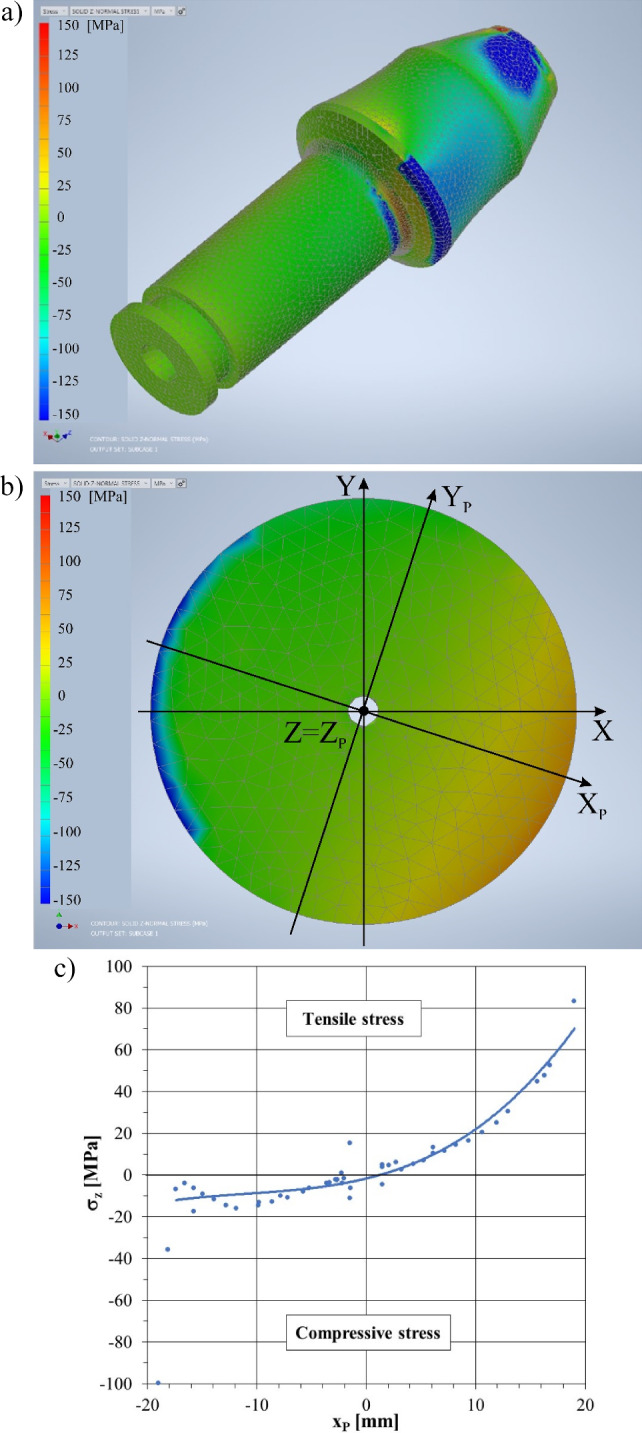
Maps of the normal stresses in the longitudinal direction (i.e., the *Z*-axis) of a pick with a monolithic structure (conventional solution): (**a**) the entire pick, (**b**) the C–C section (see Fig. 8), and (**c**) stress diagram in the plane of the bending moment on the pick shank.

For comparative purposes, the FEM model of a conventional pick with a monolithic shank is also developed (Fig. 11a). It consists of 211,278 tetrahedral solid elements based on a mesh of 305,570 nodes. The size of the mesh elements for this case is 2 mm. Within the transition radius, this mesh has a dimension of 1 mm.

#### State of static stress of the pick body subjected to the cutting load—a conventional pick and a pick equipped with a reinforcing sleeve

A complex state of stress is induced in the shank of a pick mounted in a pick holder and subjected to the action of mining forces. In this section, normal stress in the direction parallel to the Z-axis of the coordinate system associated with the pick *σ*_*Z*_ is analyzed, not von Misses stresses. The stress *σ*_*Z*_ is a measure of the effort of the tested element, and their sign indicates the areas of occurrence of compressive stresses (negative values) and particularly unfavorable tensile stresses (positive values). The distribution of normal stresses *σ*_*Z*_ on the outer surface of a conventional pick (of a monolithic structure of its body) is shown in Fig. 11a. Compressive stress concentrations occur in the pick support zones in the pick holder (the figure shows the positions at which the reaction is applied: *R*_2_ and *R*_*Z*_). These are navy blue areas on the gripping part, in the transition radius zone, and on the thrust collar. The maximum values at this position reach –834 MPa. On the other hand, in the area of the transition radius, between the gripping part and the supporting flange, a zone of increased tensile stresses is visible (orange area). Tensile stresses reach about +130 MPa here.

The distribution of stresses *σ*_*Z*_ in the cross-section of the pick shank (section C–C located in the zone of the transition radius—see Fig. 8) is largely the effect of the bending moment acting on the pick shank during mining; the method of its support affects the deformation of the gripping part of the pick. Analyzing the distribution of normal stresses in the plane of action of the bending moment X_P_Z_P_ (section C–C) (see Fig. 11b, where the axis Z_P_ coincides with the longitudinal axis of the pick Z and is perpendicular to the drawing), it can be seen that in the range of positive values of the *x*_*P*_ coordinate (right-hand side of the drawing), the gripping part of the pick shank is stretched (orange area fading to green). Tensile (positive) stresses decrease non-linearly as the pick’s longitudinal axis is approached, and then they turn into compressive (negative) stresses—see Fig. 11c. The closer to the outer surface of the pick shank, the greater these stresses (in terms of absolute value). Apart from the local stress concentration in the surface layer of the pick shank, in the contact area of its gripping part with the surface of the socket in the pick holder (within the *R*_2_ support), the maximum compressive stresses are about 4 times lower compared to the maximum tensile stresses that occur in the tested cross-section of the pick shank. This is a fairly typical picture of the stress distribution in an element set with clearance in the housing; it is subjected to an external spatial load with the dominant influence of the bending moment.

**Figure 12:**
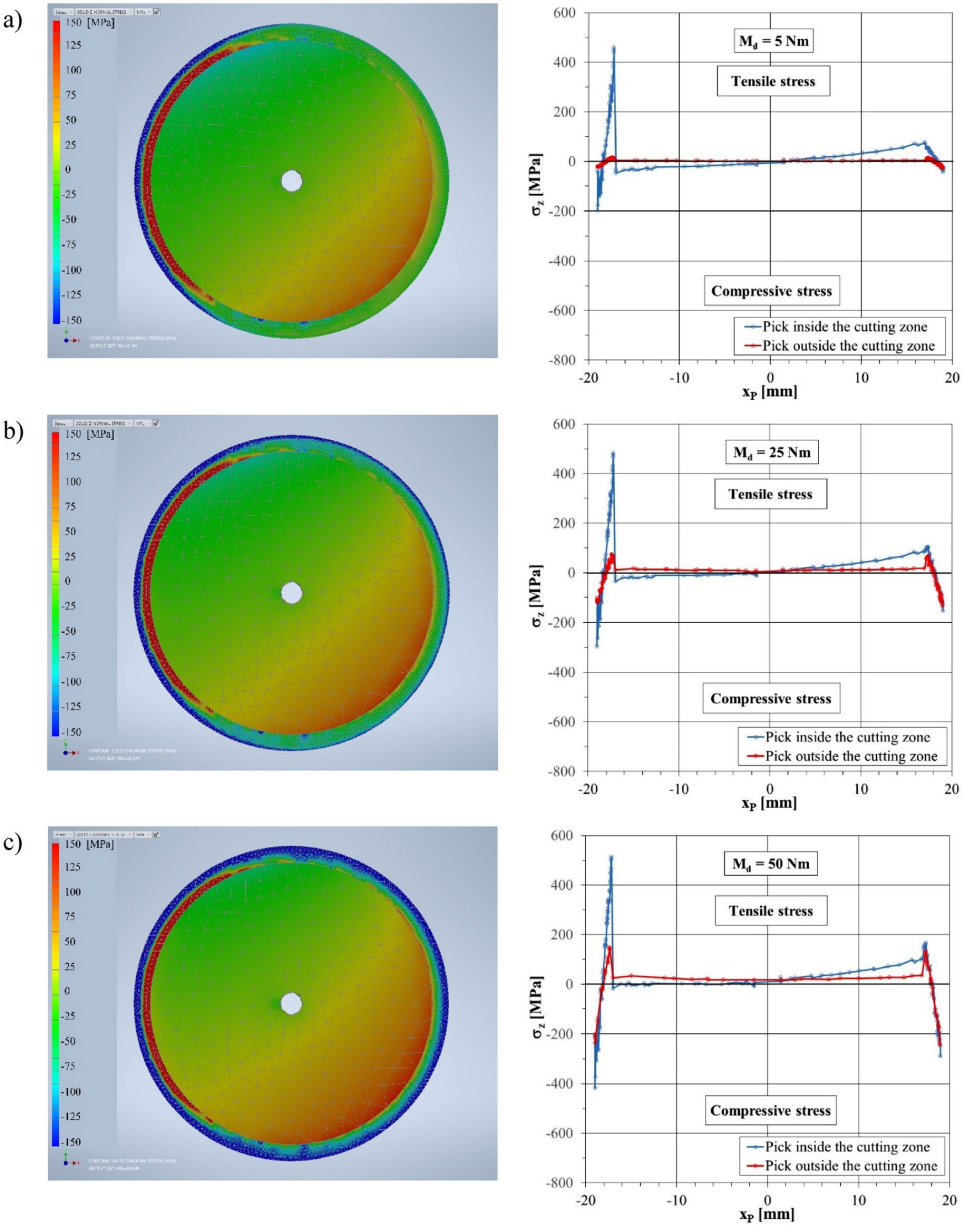

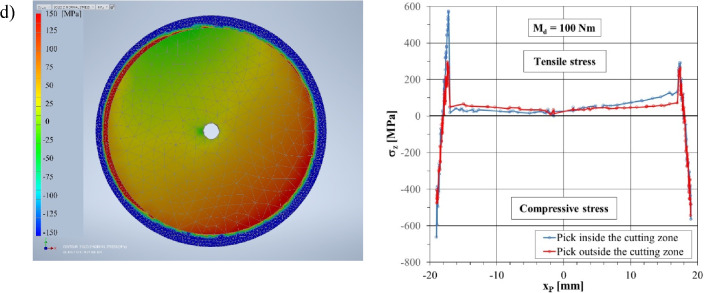
Maps of the normal stresses in the longitudinal direction (the *Z*-axis) in the C–C section of a pick equipped with a reinforcing sleeve for different tightening torque values of the tightening nut: (**a**) *M*_*d*_ = 5 Nm, (**b**) *M*_*d*_ = 25 Nm, (**c**) *M*_*d*_ = 50 Nm, and (**d**) *M*_*d*_ = 100 Nm.

The distribution of normal stresses *σ*_*Z*_ in the cross-section of the gripping part of the pick with the proposed design (equipped with a reinforcing sleeve) is different compared to the shank of a monolithic, conventional pick (Fig. 12). The stress values depend, to a large extent, on the amount of preload of the reinforcing sleeve.

Among the tested cases of loading the pick during cutting, considering the preload of the reinforcing sleeve caused by tightening the nut with the torque in the considered range, to illustrate the impact of this tension, four cases are shown in Fig. 12 that correspond to the values of the torque *M*_*d*_, which are 5, 25, 50, and 100 Nm.

The preload of the reinforcing sleeve causes a complex state of stress in the reinforcing sleeve, especially in the zone of its transition radius. In the C–C section, compressive stresses from the outer surface of the reinforcing sleeve and tensile stresses from its inner side are visible. This is illustrated by fragments of the graphs in the right-hand column of Fig. 12, marked with red lines in the coordinate ranges *x*_*P*_ ∈ 〈–19 mm, –17 mm ∧ 〈 +17 mm, +19 mm. Depending on the value of the nut tightening torque, the maximum compressive stress ranges from –38 MPa for *M*_*d*_ = 5 Nm (Fig. 12a) to as much as –560 MPa for *M*_*d*_ = 100 Nm (Fig. 12d). In turn, the maximum tensile stresses (on the inside of the reinforcing sleeve) range from + 15 MPa for *M*_*d*_ = 5 Nm to +295 MPa for *M*_*d*_ = 100 Nm. Tensile stresses are the result of the deformation of the reinforcing sleeve within the transition radius, which rests at the edge against the thrust collar of the pick shank (see Fig. 3). The pick shank is stretched by the preload force—normal stresses *σ*_*Z*_ in the C–C section have an approximately uniform distribution and range from +4 MPa for *M*_*d*_ = 5 Nm to +73 MPa for *M*_*d*_ = 100 Nm.

When the pick contacts the rock being mined, its load increases until it reaches the maximum value, which is shown in Fig. 9b. Loading the pick with cutting forces causes a change in the state of stress in its shank and the reinforcing sleeve. The lack of uniformity of the distribution of normal stresses in the cross-section of the gripping part of the pick (section C–C) is visible, caused by the action of the bending moment. However, the greater the preload of the reinforcing sleeve, the smaller this unevenness. This is especially true of the pick shank, which can be seen in the normal stress distribution maps (left-hand column of Fig. 12). As the preload increases, the yellow-orange part of the pick shank cross-section increases (tensile stresses). The reinforcing sleeve, on the other hand, becomes increasingly darker blue (compressive stresses). This is also visible in the course of normal stresses along the X_P_ axis of the tested C–C cross-section (diagrams in the right-hand column in Fig. 12 marked in blue). For example, for *M*_*d*_ = 5 Nm, the normal stresses in the reinforcing sleeve from the side of the pick support zone in the pick holder (negative values of the *x*_*P*_ coordinate) range from –199 MPa (outside the sleeve) to +458 MPa (inside the sleeve). On the other hand, on the opposite side of the tested cross-section (positive values of the *x*_*P*_ coordinate), normal stresses change on the wall thickness of the reinforcing sleeve only in the range from –44 to +71 MPa (Fig. 12a). On the other hand, for *M*_*d*_ = 100 Nm, the normal stresses in the reinforcing sleeve assume values in the range:

- from –663 to +575 MPa for negative values of the *x*_*P*_ coordinate,
- and from –565 to +292 MPa for positive values of the *x*_*P*_ coordinate.

At a small initial tension of the reinforcing sleeve (*M*_*d*_ = 5 Nm), the normal stresses in the C–C section of the pick shank along the X_P_ axis change sign (the values of these stresses range from –48 to +71 MPa). Thus, part of the cross-section is compressed (i.e., has negative values), and part is stretched (positive values)—see Fig. 12a. As the value of the reinforcing sleeve preload increases, the line representing the course of the relationship *σ*_*Z*_ = f(*x*_*P*_) moves upwards (Fig. 12b–d). When the tightening torque of the nut is 100 Nm (Fig. 12d), the pick shank is stretched over the entire surface of the C–C section, although unevenly. Normal stresses vary here from + 18 MPa for *x*_*P*_ = –17 mm to +127 MPa for *x*_*P*_ =  +17 mm.

From the point of view of the fatigue life of picks used in mining machines for cutting rocks, especially hard rocks, it is important in the outer layer zone of the gripping part of the pick (here, the reinforcing sleeve) that compressive stresses of an appropriately selected value are formed to the maximum tensile stresses from the external load^31^. As explained by the mechanism of fatigue failure of pick shanks described in ref.^31^, fatigue cracks in pick shanks subjected to variable, zero-based pulsating external loads (alternating loading and unloading of the pick) are initiated within the surface layer or directly below it. This results from the occurrence of various types of defects in their material and geometric notch that causes stress concentration. Despite the zero-based pulsating nature of the load on the picks during cutting, due to their rotation relative to the pick holder, the stresses in the gripping part of the pick have the character of a fully reversed cycle. After the cutter is rotated 180°, the part of the cross-section previously stretched by the bending moment is now compressed, and vice versa.

**Figure 13:**
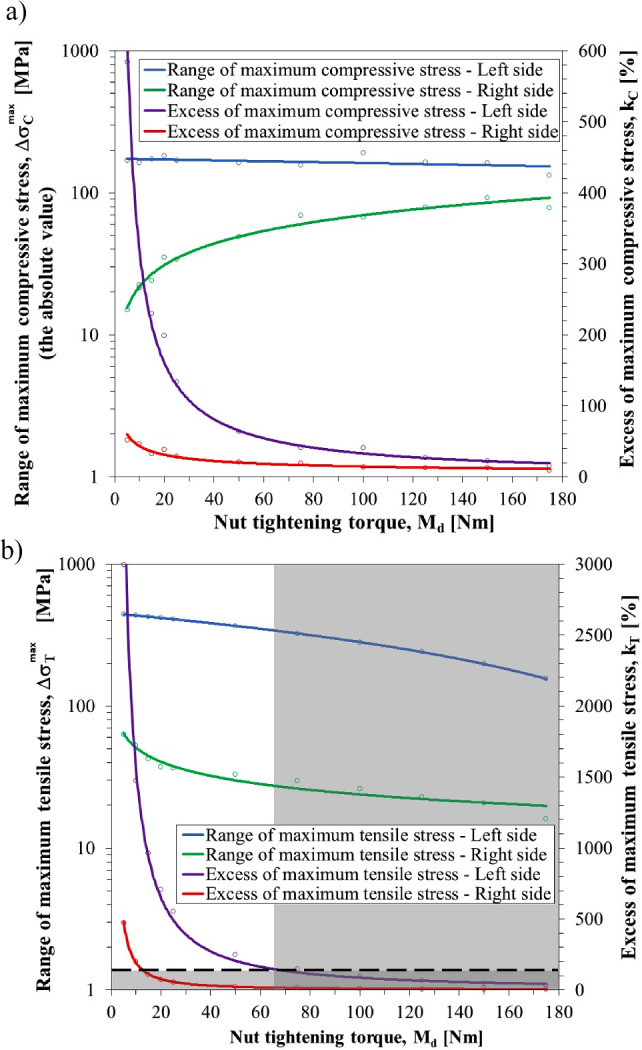
Influence of the tightening torque of the tightening nut *M*_*d*_ on (**a**) the range of variation ${\Delta \sigma }_{C}^{max}$ and the excesses of compressive stresses *k*_*C*_, and (**b**) the range of variation ${\Delta \sigma }_{T}^{max}$ and the excesses of tensile stresses *k*_*T*_ in the C–C section.

The range of variability of normal stresses in the gripping part of the pick, in successive cycles of its operation, is the difference between the stresses arising during contact with the rock being mined (loaded pick) and after its exit from the cutting zone (unloaded pick). From the side of the pick support zone in the pick holder (left-hand side of the C–C section), the impact of the tightening torque of the tightening nut on the range of variation of the maximum compressive stresses $\Delta {\sigma }_{C}^{max}$ is not too large (see Fig. 13a—blue line). In the tested range of variation of the tightening torque *M*_*d*_, i.e., from 5 to 175 Nm, the range of variation of the maximum compressive stresses ${\Delta \sigma }_{C}^{max}$ is from –133 to –192 MPa. The maximum compressive stresses during cutting (loaded pick) are in the area of 10%, which is 600% higher than the maximum values that result only from the initial tension of the reinforcing sleeve (unloaded pick). The greater the tightening torque of the tightening nut *M*_*d*_, the lower the surplus of maximum compressive stresses during mining *k*_*C*_ (purple line). The following formula expresses this surplus:

6 $$k_{C} = \frac{{\sigma_{C LP}^{max} - \sigma_{C UP}^{max} }}{{\sigma_{C UP}^{max} }} \cdot 100 = \frac{{\sigma_{C}^{max} }}{{\sigma_{C UP}^{max} }} \cdot 100$$

where *k*_*C*_ symbolizes the excess of maximum compressive stresses during cutting (%), and $\sigma_{C UP}^{max}$ and $\sigma_{C LP}^{max}$ are the maximum compressive stresses with the pick not loaded and loaded with cutting forces (MPa), respectively.

This appears different on the right-hand side of the cross-section, which in the case of a pick with a conventional design (monolithic shank) is stretched (cf. Fig. 11c). The range of variability of the maximum compressive stresses $\sigma_{C}^{max}$ increases with the tightening torque of the tightening nut in the range from –15 to –92 MPa (green line). The maximum value of the compressive stresses during mining (loaded pick) is higher than the maximum value caused by the initial tension of the reinforcing sleeve (unloaded pick) by only 10–50% (red line). Similar to the left-hand side of cross-section C–C, the surplus of the maximum compressive stresses *k*_*C*_ (occurring in the reinforcing sleeve for the loaded and unloaded pick) decreases with the tightening torque of the tightening nut *M*_*d*_.

The range of variability of the maximum tensile stresses $\Delta \sigma_{T}^{max}$ in the analyzed cross-section of the reinforcing sleeve, occurring on its inner side, decreases with the tightening torque value of the tightening nut *M*_*d*_ (Fig. 13b). This descent is particularly evident in the left-hand part of the C–C section, from the side of the pick support zone in the pick holder (blue line). With an increase in the value of the tightening torque in the range from 5 to 175 Nm, the range of the variability of these stresses decreases from +443 to +156 MPa, i.e., almost 3 times. For *M*_*d*_ ≥ 100 Nm, the surplus of the maximum tensile stresses *k*_*T*_ for the loaded pick during mining to the maximum values of these stresses for the unloaded pick does not exceed 95% (purple line). This surplus is expressed by the formula:

7 $$k_{T} = \frac{{\sigma_{T LP}^{max} - \sigma_{T UP}^{max} }}{{\sigma_{T UP}^{max} }} \cdot 100 = \frac{{\sigma_{T}^{max} }}{{\sigma_{T UP}^{max} }} \cdot 100$$

where *k*_*T*_ signifies the excess of maximum tensile stresses during cutting (%), and ${\sigma }_{T UP}^{max}$ and ${\sigma }_{T LP}^{max}$ are the maximum tensile stresses (MPa) with pick not loaded and loaded with cutting forces, respectively.

A fairly high variability of the maximum tensile stress on the inside of the reinforcing sleeve in the pick support zone in the pick holder during cyclic loading and unloading of the pick is not dangerous because the sleeve should be made of steel with higher (compared to the pick shank) mechanical properties after thermo-chemical treatment. As a result of this treatment, the state of compressive residual stresses in the reinforcing sleeve is induced (as a result of the increase in the specific volume and the growth in the dislocation density of the treated layer). The preload makes the system even more effective.

The range of variability of the maximum tensile stresses ${\Delta \sigma }_{T}^{max}$ that occur in the right-hand part of the C–C section, and especially in the reinforcing sleeve, is smaller compared to the one described above (see Fig. 13b—green line). With an increase in the tightening torque of the tightening nut, this range decreases by 4 times, because it is in the range from +63 to +16 MPa. The maximum values of tensile stresses for a pick loaded with cutting forces are from 4 to 470% higher than the maximum values of these stresses for an unloaded pick. The excess of the maximum tensile stresses *k*_*T*_ decreases with an increase in the value of the moment *M*_*d*_ (red line). The introduction of a not-very-high preload improves the state of the pick’s shank effort on the stretching side. For example, for *M*_*d*_ = 5 Nm, the range of variability of the maximum tensile stresses in this part of the tested cross-section of the pick decreased by about 15% compared to the conventional pick (cf. Fig. 11b).

**Figure 14:**
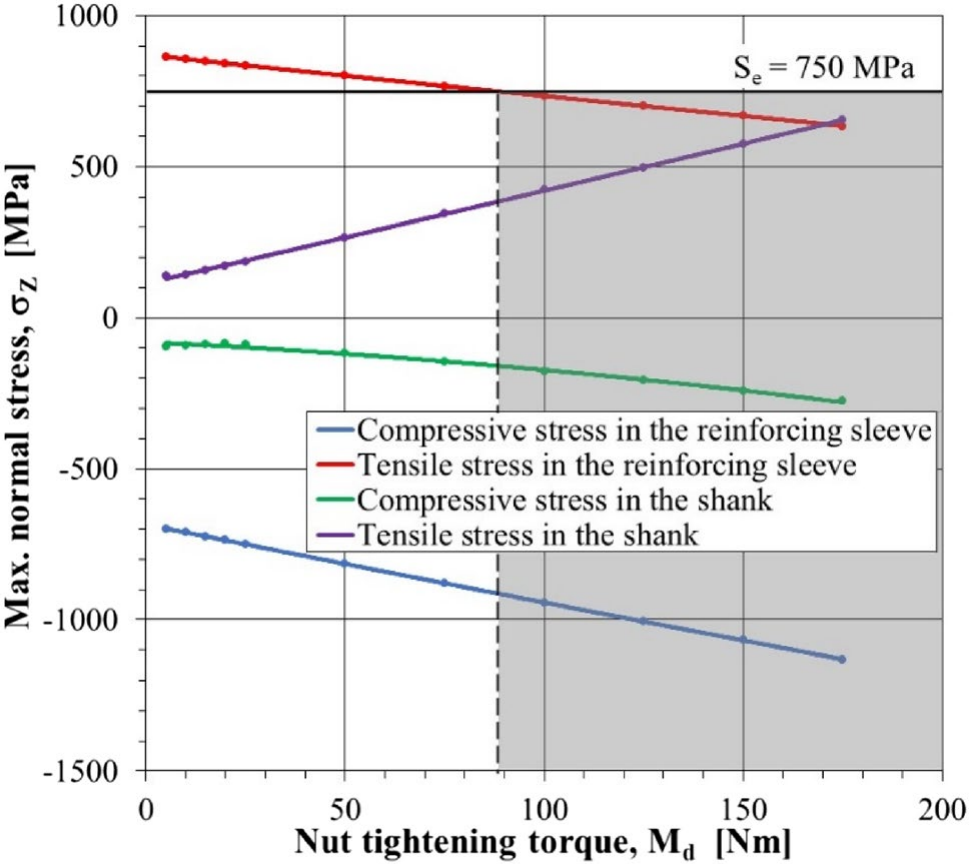
Influence of the tightening torque of the tightening nut *M*_*d*_ on the maximum values of normal stresses in the direction of the *Z*-axis in the reinforcing sleeve and the pick shaft.

Figure 14 shows the effect of tightening torque on the tightening nut of the peak values of tensile and compressive stresses in the pick shank (purple and green lines, respectively) and the reinforcing sleeve (red and blue lines, respectively). With an increase in the value of this moment, the maximum tensile stresses in the pick shank grow from +139 to +654 MPa, i.e., by nearly 5 times. The maximum value of the compressive stresses in this element also increases (the absolute value too). In the considered tightening torque range, the maximum compressive stresses change in the range from –95 to –276 MPa, i.e., nearly by 3 times. An increase in the preload caused by tightening the nut with an increasing value of the torque *M*_*d*_ results in a rise in the compressive stresses in the reinforcing sleeve. In the range of the tightening torque of the tightening nut that varies from 5 to 175 Nm, the maximum compressive stresses in this element vary from –700 to –1130 MPa (i.e., by 60%). The effect of the tightening torque of the tightening nut on the tensile stresses in the reinforcing sleeve is different. An increase in the value of the tightening torque in the tested range causes a decrease in the maximum value of these stresses in the range from +862 to +634 MPa, i.e., by about 25%.

The purpose of the introduced structural modifications of the pick is to reduce the range (the amplitude) of the variability of the tensile stresses in its gripping part, especially in the zone of the transition radius. These stresses are particularly dangerous from the point of view of the fatigue life of the cutting picks. It is also advantageous when the fully reversed cycle of the stress variation in the pick shank is as close as possible to one-way character. This effect can be achieved by preloading the elements that compose the gripping part of the pick. Simulation tests of the state of stress, and the effect of the tightening torque of the tightening nut on it, indicate that a properly selected value of the tightening torque *M*_*d*_, determining the value of the initial tension force of the reinforcing sleeve *Q*_*d*_, should:

- ensure the minimization of the range of variation (the amplitude) of the maximum tensile stresses ${\sigma }_{T}^{max}$ in the gripping part of the pick, which is tantamount to minimizing the excess of maximum tensile stresses during cutting *k*_*T*_ over the value resulting from the initial tension,
- not allow an exceeding of the maximum due to fatigue strength, tensile stresses caused by the initial tension, and the load on the pick during mining:
  8 $$\left.\begin{array}{c}{k}_{T}\to min\\ \\ {\sigma }_{T}^{max}\le {S}_{e}=0.45\cdot {S}_{u}\end{array}\right\} \Rightarrow {M}_{d}$$

where *S*_*e*_ is the endurance limit (MPa), and *S*_*u*_ denotes the ultimate strength of the material from which the pick elements are made (MPa).

The analysis of the parameters characterizing the course of cyclically varying tensile stresses in cross-section C–C with the maximum value (Fig. 13a) shows that the fulfillment of the first of the listed criteria is possible when the tightening torque of the tightening nut *M*_*d*_ is greater than 65 Nm. However, comparing the maximum tensile stresses with the endurance limit for the adopted steel grade, it can be concluded that the fulfillment of the second of the above conditions is possible for *M*_*d*_ ≥ 90 Nm. Nevertheless, too high a value of this moment causes an increase in tensile stresses to values close to the fatigue strength of the steel from which the pick shank is made (purple line in Fig. 14). With this in mind, the tightening torque of the tightening nut *M*_*d*_ = 100 Nm is finally adopted.

## Summary and conclusions

The system of hydrodynamic rotation of picks around the longitudinal axis presented in this article, integrated with a two-stage central spraying during rock cutting, is characterized by the high efficiency of the sprinkling process and the tangential-rotary pick rotation. It enables a significant improvement in its service life by increasing the resistance to the destructive processes of picks that occur in practice, such as abrasive and erosive wear, fretting, and thermal fatigue of cutting pick tips; these factors may significantly enhance the susceptibility to fatigue cracking of pick shanks. The use of this solution enables a substantial reduction in the consumption of spraying water and energy savings for the execution of the mining process. The system of hydrodynamic rotation of picks combined with effective spraying is particularly suitable for use with cutting picks with ring-type tips. It can also be employed for other types of tangential-rotary picks, for example, crown picks^22^. This solution can easily be adapted for use in machines for milling surfaces of roads, squares, and airport runways in the process of their renovation. It makes it possible to reduce the generation of rock dust in the mining process, including the respirable dust absorbed into the respiratory tract, which is particularly dangerous to human health. The described system is also characterized by high ecological values.

Due to the efficiency of this process, the current research of the authors of this article relates to the selection of the optimal geometrical features of the hydrodynamic pick rotation mechanism. The results of these studies will be presented in subsequent publications.

The developed solution of the tangential-rotary pick with a new design is characterized by high repair susceptibility. After the picks are removed from service due to their excessive wear, many elements can be reused. In particular, reinforcing sleeves, tightening nuts, and elements of the hydrodynamic rotation of picks and two-stage sprinkling can be redeployed many times. Disassembly operations for overhaul and reassembly of parts after their verification can be performed in typical mechanical workshop conditions of the user of mining machines. This allows for additional large technical and economic effects.

Extensive simulation studies performed using the finite element method enable knowledge of the distribution of stresses in the elements of the pick of the proposed design and its comparison with the stresses that occur in a conventional pick loaded in the same way with forces due to rock cutting with given strength parameters. The analysis of the state of stress enables the following conclusions:

- Even with relatively small values of the preload of the reinforcing sleeve in the pick, according to the solution, the state of stress in the gripping part of the pick is improved, especially in the areas of tensile stresses, particularly undesirable due to fatigue failure.
- The induction of a relatively small preload of the reinforcing sleeve, as a result of tightening the tightening nut with a torque of *M*_*d*_ = 5 Nm, causes a decrease by about 15% of the range of variation of the maximum tensile stresses in the pick’s shank in the considered critical cross-section compared to a conventional pick (with 83–71 MPa). With higher tightening torques, this reduction is even greater.
- The results of the model tests were used to determine the conditions for selecting the tightening torque of the tightening nut. Based on these conditions, an advantageous (from the point of view of fatigue strength) value of the tightening torque was selected for the given structural form of the pick, the assumed material properties, and the size of its load. Assuming that the maximum resultant load of the pick during the cutting does not exceed 38 kN, the tightening torque of the tightening nut should preferably be greater than 90 Nm. The maximum tensile stresses in the reinforcing sleeve are then the endurance limit (for the steel used during the tests). However, the value of the tightening torque of the reinforcing nut cannot be too high because, with its increase, the maximum tensile stresses in the pick shank ascend. With overly high values for the torque *M*_*d*_, there is a risk of exceeding the fatigue strength of the material from which the knife shaft is made.
- For the adopted assumptions, it was therefore concluded that the value of the tightening torque of the tightening nut should preferably be 100 Nm. In this way, a reduction of over 30% in the range of variation of tensile stresses that occur in the zone of the transition radius of the pick shank is obtained compared to a conventional pick (the range of variation of tensile stresses here is 89 MPa and, for a pick with a monolithic shank, it reaches 130 MPa). It is expected that this will contribute to a significant (even four-fold) increase in the fatigue life of the tangential-rotary picks.

## Data Availability

The datasets used and/or analyzed during the current study available from the corresponding author on reasonable request.
